# A proposed closed-loop framework for postoperative management of OVCF integrating biosensor-enabled monitoring and traditional Chinese medicine formulas: from dynamic assessment to precision intervention

**DOI:** 10.3389/fphar.2026.1758723

**Published:** 2026-03-05

**Authors:** Chuxi Wang, Ziwei Yu, Yingzi Liao, Jiafang Zhang

**Affiliations:** 1 Second Clinical Medical College, Fujian University of Traditional Chinese Medicine, Fuzhou, China; 2 Department of Spinal Surgery, The Second Affiliated Hospital of Fujian Traditional Chinese Medical University, Fuzhou, China

**Keywords:** biosensing technique, bone turnover markers, Chinese herbal compound, osteoporotic vertebral compression fracture, precision medicine

## Abstract

Postoperative management of osteoporotic vertebral compression fracture (OVCF) remains challenging because conventional bone mineral density (BMD) and imaging assessments are intrinsically delayed, while bone turnover markers (BTMs) that reflect real-time remodeling dynamics are difficult to measure frequently in routine care. In parallel, commonly used anti-osteoporotic medications may be limited by delayed onset of measurable response and concerns regarding long-term tolerability in certain populations. This article is a narrative review and conceptual perspective that synthesizes recent advances in biosensor-enabled point-of-care testing—highlighting organic optoelectrochemical transistors (OPECT)—and mechanistic pharmacology evidence for kidney-tonifying and blood-activating traditional Chinese medicine (TCM) formulas. On this basis, we propose a closed-loop framework that links high-frequency BTM monitoring to biomarker-informed optimization of postoperative integrative management. Importantly, this work does not report original clinical implementation data; the proposed framework is intended to guide future translational research, standardization, and prospective clinical validation.

## 1 Introduction

The phenomenon of osteoporotic vertebral compression fractures (OVCF) is a subject of increasing concern in the context of the global population ageing. There is a demonstrable increase in the incidence of this condition. The resulting pain, deformity, and functional impairment render it a formidable public health challenge (Xu et al., 2024; Zhang B. et al., 2025). Although minimally invasive techniques such as percutaneous vertebroplasty (PVP) can effectively stabilise the injured vertebrae and alleviate acute pain, they do not address the fundamental cause of systemic bone metabolic imbalance. Consequently, patients remain at extremely high risk of subsequent fractures following treatment (Liu et al., 2025; Marselou et al., 2025).

The clinical trajectory for managing osteoporotic vertebral fractures (OVCF) post-surgery is currently obstructed by a persistent dual bottleneck. At the therapeutic level, the reliance on conventional anti-osteoporotic agents remains problematic, as these pharmacological options often struggle with suboptimal onset speeds and questionable long-term safety profiles (Garton, 2020). This therapeutic gap is further exacerbated by a systemic failure in assessment: traditional diagnostic pillars—namely bone mineral density (BMD) and radiological imaging—function as “lagging indicators” that fail to capture the immediate, fluid shifts in bone physiology. While biochemical markers of bone turnover (BTMs) theoretically provide a window into real-time bone remodeling dynamics, their clinical utility has been largely stifled. The logistical friction of current testing protocols, combined with a lack of viable point-of-care implementation, has relegated BTMs to the periphery of routine monitoring (Cosman et al., 2023; Wiklund et al., 2024). Consequently, there is a pressing clinical exigency to move beyond these static frameworks. We propose a paradigm shift toward integrated Chinese and Western medical strategies capable of rapid bone metabolism modulation, underpinned by a responsive, dynamic efficacy assessment system that bridges the gap between biological potential and clinical oversight.

In this context, biosensor-enabled high-frequency monitoring of bone metabolism, together with mechanism-informed traditional Chinese medicine (TCM) approaches rooted in the theory that “the kidneys govern the bones,” represents a promising direction to address current gaps in postoperative care. Here, we synthesize relevant advances and propose a translational, closed-loop conceptual framework integrating “dynamic monitoring” with “precision intervention” for postoperative management of osteoporotic vertebral compression fracture (OVCF). This article is a review and does not present original clinical trial data or outcomes.

## 2 How novel biosensing technologies are reshaping dynamic monitoring systems for bone metabolism

### 2.1 The value of dynamic monitoring for bone turnover markers (BTMs) and the clinical disconnect with traditional detection methods

Biochemical markers of bone turnover (BTMs), such as the N-terminal propeptide of type I procollagen (PINP), reflecting bone formation, and the cross-linked C-terminal telopeptide of type I collagen (β-CTX), characterising bone resorption, are lauded by the international academic community as “windows into bone metabolic dynamics” (Schini et al., 2023). In comparison with static bone mineral density (BMD), BTMs have been shown to exhibit significant changes within weeks to months following effective anti-osteoporosis treatment. This offers high sensitivity for the assessment of early treatment response, monitoring of patient compliance, and prediction of long-term fracture risk (Bertoldo et al., 2024). Large-scale clinical studies have confirmed that early alterations in BTMs can effectively predict subsequent BMD improvement and fracture risk reduction (Eastell et al., 2025).

Despite the clinical promise of dynamic BTM assessment, its transition from bench to bedside remains hamstrung by significant systemic barriers. The prevailing diagnostic landscape is still dominated by centralized laboratory platforms—primarily enzyme-linked immunosorbent assay (ELISA) and electrochemiluminescent immunoassay (ECLIA). These methodologies, while precise, are fraught with operational friction; they necessitate specialized technical oversight, prohibitively expensive instrumentation, and cumbersome processing cycles that stretch turnaround times from hours into days (Cavalier et al., 2016). Such logistical bottlenecks effectively preclude the deployment of immediate, point-of-care testing (POCT) within the fast-paced environments of orthopedic outpatient clinics or community-based recovery settings. This “technological lag” creates a diagnostic vacuum, preventing BTMs from evolving into the high-frequency, personalized follow-up tools required for post-operative OVCF management. Consequently, the intrinsic clinical utility of these biomarkers is frequently diluted, as they fail to provide the real-time actionable insights necessary for truly individualized patient care (Wu et al., 2025).

### 2.2 Principles of biosensing technology and early exploration in bone metabolism monitoring

Biosensing technology is regarded as an ideal platform for point-of-care testing (POCT) due to its outstanding advantages of high sensitivity, high specificity, rapid response, ease of operation, and suitability for miniaturisation (Yoon et al., 2022; Hosseini Aghouzi et al., 2025). As an analytical device that converts biological recognition events into quantifiable electrical/optical signals, biosensing technology is an effective tool for medical analysis. A biosensor is a device that can be used to detect specific biological substances. It is made up of two parts: a biosensor element, which can be anything from antibodies to enzymes, and a signal transducer, which can be used to convert the biosensor’s signal into an observable signal. This allows the biosensor to detect specific substances in a sample quickly and easily (Zhang, 2023).

In the domain of bone research, scholars have long explored the application of biosensor technology. In the early stages of research, the development of electrochemical sensors was the primary focus. These sensors were based on the catalytic activity of alkaline phosphatase (ALP), with the intention of indirectly assessing osteoblast activity (Sanchez et al., 2020). In the field of biosensing, other teams have explored the use of nucleic acid aptamers or molecularly imprinted polymers as recognition elements in constructing sensors that target specific markers, such as osteocalcin (OC). These sensors have exhibited good specificity in model solutions (Jiang et al., 2025; Li et al., 2025). However, the majority of these early explorations remain confined to laboratory validation stages. When confronted with complex real-world clinical samples (e.g., serum or urine), their detection performance often faces significant challenges in terms of interference resistance, long-term stability, and practicality. Moreover, there is a paucity of clinical reports on the systematic integration and successful application of such sensors for dynamic monitoring of patients following OVCF surgery.

### 2.3 OPECT technology pioneers a new era in bone metabolism monitoring

To transcend the performance ceilings inherent in conventional sensing modalities, Organic Optoelectrochemical Transistors (OPECT) have emerged as a formidable biosensing architecture, predicated on a unique signal transduction paradigm (Ju et al., 2023; Zhang J. M. et al., 2025). Central to the OPECT’s efficacy is a photosensitive organic semiconductor channel that orchestrates a sophisticated tripartite conversion—an integrated “biological-to-optical-to-electrical” cascade. Rather than a simple linear relay, this mechanism begins at the molecular interface, where discrete biorecognition events—such as the high-affinity binding between aptamers and target BTMs—induce subtle perturbations in the optical field. These optical modulations are not merely recorded but are actively transformed and amplified through the inherent transconductance of the organic semiconductor framework. By leveraging the transistor’s gate-controlled gain, the OPECT architecture translates minute biochemical flux into robust, high-fidelity electronic signatures, yielding a detectable output that far exceeds the sensitivity thresholds of non-amplified platforms.

The integration of bio-recognition, transistor signal amplification, and optoelectronic detection with low-background noise has been demonstrated to yield superior performance in comparison to conventional sensors. Research indicates (Liu et al., 2024) that aptamer-functionalised OPECT sensors achieve detection limits at the pg/mL level for multiple biomarkers, exhibiting sensitivity that far surpasses that of traditional methods. At present, the primary focus of state-of-the-art applications of OPECT technology is on environmental monitoring (e.g., heavy metal ion detection) and certain disease markers (e.g., prostate-specific antigen) (Yuan et al., 2024; Qu et al., 2023).

Extending OPECT-based biosensing platforms to bone metabolism monitoring is an active area of exploration. In principle, the high sensitivity and low-noise signal amplification of OPECT devices could enable the quantification of low-abundance BTMs (e.g., NTX and osteocalcin) from minimally invasive samples such as urine, thereby supporting more frequent monitoring outside centralized laboratories. However, current evidence for BTM-oriented OPECT applications remains at an early, proof-of-concept stage, and analytical validation in complex clinical matrices as well as prospective clinical utility studies will be required before broad implementation can be considered.

## 3 The unique value of kidney-tonifying and blood-activating Chinese herbal formulas in postoperative systemic regulation following OVCF surgery

In postoperative OVCF management, Traditional Chinese Medicine (TCM) conceptualizes the pathogenesis based on the principles that “the kidneys govern the bones and produce marrow” and “qi stagnation and blood stasis” (Wang et al., 2022). This framework interprets OVCF as a condition in which kidney essence deficiency constitutes the underlying susceptibility, while qi stagnation and blood stasis contribute to pain, impaired microcirculation, and delayed repair. Accordingly, the commonly proposed therapeutic principle is to “tonify the kidneys and strengthen bones” while “activating blood circulation and resolving stasis.” Importantly, in the context of this review, such statements are presented as a theoretical and historical rationale for potential adjunctive, integrative strategies, rather than evidence that a standardized regimen has been widely implemented with confirmed benefit in routine postoperative practice.

Contemporary pharmacological studies have begun to clarify plausible biological bases for this principle. For example, kidney-tonifying herbs such as Morinda officinalis and Epimedium sagittatum have been reported to modulate pathways including Wnt/β-catenin, thereby supporting osteogenic differentiation while attenuating osteoclastogenesis in experimental models (Li et al., 2023; Zhang et al., 2018; Tripathi et al., 2022). Meanwhile, blood-activating herbs such as Salvia miltiorrhiza and Panax notoginseng may improve the repair microenvironment via microcirculatory support and anti-inflammatory actions, suggesting a multi-target regulatory pattern involving “promotion of bone formation–suppression of bone resorption–optimization of the bone microenvironment” (Zhu et al., 2025; Yang et al., 2025; Yin et al., 2023).

A limited number of clinical reports have explored adjunctive use of related formulas after vertebral augmentation, with some studies describing improvements in pain/function measures and bone metabolism–related outcomes (Sharma et al., 2023; Huang et al., 2024). However, heterogeneity in study design, formula composition, and outcome reporting warrants cautious interpretation, and higher-quality prospective trials are still needed—particularly for hard endpoints such as refracture. Therefore, rather than implying established clinical effectiveness, this review emphasizes that objective biomarkers capable of reflecting early remodeling dynamics may provide a quantitative window to test mechanistic hypotheses and to support future standardization of integrative postoperative strategies.

Notably, “rapid onset” in postoperative osteoporosis management should be interpreted in a measurable pharmacodynamic sense—i.e., how early an intervention produces reproducible changes in BTMs (e.g., percent change in PINP and β-CTX/NTX) and/or clinically meaningful symptom trajectories (e.g., pain and function) within predefined windows (such as postoperative weeks 2–4) (Oršolić et al., 2018; Lai et al., 2025). Conventional anti-osteoporotic agents may show delayed visibility on BMD or imaging, whereas earlier biomarker shifts can precede structural endpoints. In this context, kidney-tonifying and blood-activating TCM formulas may plausibly yield earlier composite benefits in selected patients by simultaneously modulating inflammatory signaling, microcirculation, and remodeling-related pathways; these dimensions may manifest earlier as improvements in pain/function and short-term biomarker trajectories. However, the magnitude and consistency of any “earlier benefit” relative to standard pharmacotherapy remains insufficiently established, and should be tested in prospective, controlled studies with prespecified early endpoints. Accordingly, integrating high-frequency BTM monitoring is proposed as a way to quantify early response, prevent overinterpretation based on delayed BMD changes, and enable more transparent comparison across therapeutic strategies.

### 3.1 Biomarker–constituent–mechanism mapping to support dynamic monitoring

To strengthen the correlation between monitored BTMs and the pharmacology of kidney-tonifying and blood-activating formulas, we summarize representative associations among key biomarkers, bioactive constituents, signaling pathways, and the expected direction of change. This mapping is intended as a mechanistic rationale for biomarker-guided optimization and should be refined as higher-quality clinical and translational evidence accumulates.

Importantly, the BTMs selected for dynamic monitoring are not arbitrary: they are proximal pharmacodynamic readouts of the very remodeling axes targeted by the mapped bioactive constituents in Table 1 (i.e., osteoclast activity/resorption reflected by β-CTX/NTX versus osteoblast-driven formation reflected by PINP/BALP/OC). Therefore, linking “marker trajectories” to “compound–pathway modules” provides a mechanistically anchored rationale for the proposed closed-loop adjustment of formula orientation.

**TABLE 1:** Representative biomarker–constituent–mechanism mapping for kidney-tonifying and blood-activating TCM formulas, summarizing plausible links between monitored BTMs, key constituents, signaling pathways, expected biomarker directionality, and evidence type (preclinical vs. clinical).

BTM	Biological meaning	Representative herbs/formulas	Key constituents	Putative pathways	Expected BTM direction (early window)	Evidence type (preclinical/clinical)
PINP/BALP	Bone formation	Kidney-tonifying formulas (Epimedium, Morinda)	Icariin; anthraquinones	Wnt/β-catenin; osteoblast differentiation	↑	*In vitro*/*in vivo*; limited clinical
Osteocalcin (OC)	Formation/turnover	Kidney-tonifying + blood-activating combinations	Icariin; notoginsenosides	Runx2/OSX; PI3K–Akt (reported)	↑	*In vitro*/*in vivo*; proof-of-concept
β-CTX	Bone resorption	Kidney-tonifying formulas; integrative regimens	Icariin; Morinda constituents	RANKL/OPG; NF-κB	↓	*In vivo*; some clinical
NTX	Bone resorption	Blood-activating adjuncts; integrative regimens	Tanshinone IIA; notoginsenosides	Inflammation/microcirculation pathways (NF-κB; VEGF)	↓	*In vivo*; limited clinical
PINP + β-CTX trajectory	Formation–resorption balance	Closed-loop optimization (dose/duration/formula modification)	Multi-component	Multi-pathway regulation	Trend-dependent	Conceptual framework

The associations listed are intended as hypothesis-supporting links for biomarker-guided optimization rather than definitive causal proof. “Expected BTM, direction” refers to the anticipated early trajectory under a favorable response (e.g., weeks 2–4 to 8–12), which should be confirmed in prospective studies.

## 4 Integration and outlook: establishing a new closed-loop management paradigm of “dynamic monitoring-precision intervention”

**FIGURE 1:**
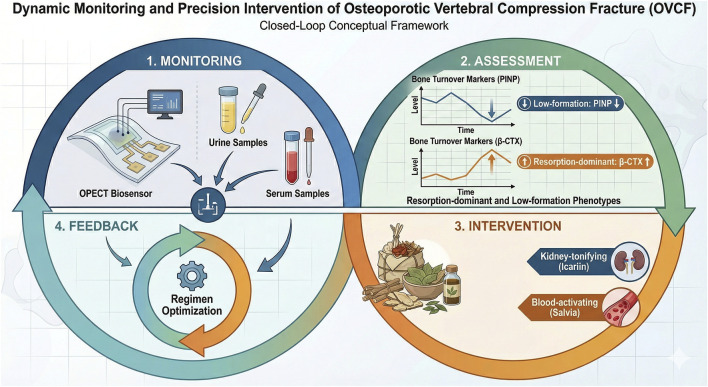
The Closed-Loop Framework of “Dynamic Monitoring–Precision Intervention” for Post-OVCF Management. This conceptual framework integrates cutting-edge organic optoelectrochemical transistor (OPECT)-enabled biosensing with mechanism-informed Traditional Chinese Medicine (TCM) to optimize postoperative care for osteoporotic vertebral compression fracture (OVCF). The system operates through four interconnected stages: (1.) Dynamic Monitoring: High-frequency measurement of key bone turnover markers (BTMs)—including PINP, β-CTX, NTX, and Osteocalcin—is facilitated by OPECT-based point-of-care testing (POCT) platforms. These devices enable rapid, high-sensitivity quantification from minimally invasive samples (e.g., urine or serum) outside of centralized laboratories. (2.) Assessment and Phenotyping: Longitudinal biomarker trajectories are analyzed to identify specific postoperative response phenotypes rather than relying on single time-point values. Key categories include resorption-dominant patterns (persistently elevated resorption markers), low-formation responses (blunted rise in formation markers), and high-turnover mixed states. (3.) Precision Intervention: Therapeutic regimens may be explored in a biomarker-informed manner within prospective study protocols. Under the principle of “tonifying the kidneys and activating blood,” formula orientations are adjusted; for instance, resorption-dominant patterns may prioritize blood-activating and anti-inflammatory components (e.g., *Salvia miltiorrhiza*), while low-formation responses emphasize pro-osteogenic, kidney-tonifying constituents (e.g., *Epimedium* or *Morinda officinalis*). (4.) Iterative Feedback: The “monitoring–assessment–intervention–re-monitoring” loop provides a continuous feedback structure for regimen refinement and hypothesis testing. The entire workflow is supported by robust data governance, ethical principles, and clinical accountability to ensure patient safety and data privacy.

Drawing upon the above two pillars, we propose a closed-loop conceptual framework termed “Dynamic Monitoring–Precision Intervention.” The core idea is to integrate biosensor-enabled, high-frequency monitoring of key BTMs with the multi-target regulatory features of kidney-tonifying and blood-activating TCM formulas. A hypothesis-generating implementation pathway could be considered as follows: (1) during monitoring, point-of-care biosensing may facilitate more convenient and more frequent measurement of BTMs (e.g., NTX, osteocalcin) from minimally invasive samples such as urine; (2) during assessment, biomarker trajectories could help characterize early pharmacodynamic responses and inter-individual variability; (3) during intervention, regimen optimization (e.g., dose, duration, and formula modification) might be explored in a biomarker-informed manner within prospective study protocols; and (4) iterative monitoring may provide a feedback structure to support hypothesis testing and future standardization. Importantly, this framework is proposed for translational development and requires prospective validation before it can be recommended as a routine clinical pathway (Figure 1).

### 4.1 From dynamic monitoring to precision intervention: a biomarker-guided decision framework

To operationalize “precision intervention,” dynamic monitoring should be linked to predefined decision points rather than interpreted as descriptive data alone. We propose a biomarker-guided decision framework that can be refined in future prospective studies and adapted to local practice patterns. Monitoring panel and time windows: Key BTMs (e.g., PINP for formation and β-CTX/NTX for resorption, with optional osteocalcin) may be measured at baseline (postoperative week 0), early follow-up (weeks 2–4), consolidation (weeks 8–12), and thereafter according to clinical needs. Emphasis should be placed on within-patient trajectories and percent change over time, rather than single time-point values. Response phenotypes based on trajectories: (i) resorption-dominant pattern (persistently elevated resorption markers or insufficient suppression), (ii) low-formation response (blunted rise in formation markers during recovery), (iii) high-turnover mixed pattern (both formation and resorption markers elevated and/or highly variable), (iv) slow responder/stable low-turnover (minimal change in both domains over time), and (v) clinical–biomarker discordance (pain/function trajectory inconsistent with BTM trends). These phenotypes are intended as pragmatic categories for hypothesis generation and should be validated against clinical outcomes.

Linking phenotypes to intervention hypotheses (to be validated). For a resorption-dominant pattern, an intervention hypothesis is to prioritize strategies that enhance anti-resorptive balance and microenvironment stabilization (e.g., considering formula orientations emphasizing blood-activating/anti-inflammatory components alongside standard-of-care pharmacotherapy when indicated), followed by early re-monitoring to confirm suppression trends. For a low-formation response, the hypothesis is to emphasize osteogenic support and recovery-stage consolidation (e.g., kidney-tonifying orientations and rehabilitation synergy), while ensuring adequate nutritional and endocrine evaluation. For a high-turnover mixed pattern, the hypothesis is to adopt a balanced, safety-first approach (moderate resorption-modulating + osteogenesis-supporting + anti-inflammatory/oxidative-stress buffering) and to actively rule out confounders (e.g., acute inflammation, endocrine drivers, medications) before escalating any single-direction strategy. For slow responders/stable low-turnover, the hypothesis is to maintain the baseline regimen with stepwise refinement focused on adherence, comorbidity management, and non-pharmacologic recovery supports, while extending the monitoring window before judging non-response. For clinical–biomarker discordance, the hypothesis is to avoid marker-only escalation and instead perform a holistic reassessment (comorbidities, sampling/assay factors, medications, and syndrome differentiation), repeating BTMs to confirm reproducible trends prior to modifying the regimen. In all scenarios, BTM-guided decisions should be treated as decision support within prospective protocols, and any regimen modification should be coupled with predefined re-monitoring intervals and safety surveillance (Table 2).

**TABLE 2:** Example biomarker-guided decision matrix linking BTM trajectories to potential postoperative integrative management adjustments.

Phenotype (BTM trajectory)	Operational definition (example thresholds^a^)	Mechanism-informed TCM modules/orientation	Conventional care considerations	Re-monitoring and decision trigger
Resorption-dominant (insufficient suppression)	β-CTX/NTX remains above baseline or fails to decline by ∼20–30% within 4–8 weeks	Emphasize modules targeting osteoclastogenesis/resorption (e.g., RANKL–NF-κB axis) and inflammation–oxidative stress, optionally combined with microcirculation support; maintain a supportive kidney-tonifying background module	Check adherence; Ca/Vit D; renal function; secondary causes; optimize anti-resorptives per guidelines when indicated	Recheck BTMs in 2–4 weeks; trigger adjustment if β-CTX/NTX rebounds or symptoms worsen
Low-formation response (blunted repair signal)	PINP/BALP (±OC) fails to rise or remains low over 4–12 weeks despite resorption control	Prioritize modules supporting osteoblast differentiation/osteogenesis (e.g., Wnt/β-catenin, BMP/TGF-β–Smad) and matrix formation; consider adding microcirculation/anti-inflammatory module if healing is impeded by pain/inflammation	Evaluate nutrition/protein; mobility/rehab; endocrine status; consider anabolic/formation-support strategies when indicated	Recheck in ∼4 weeks; trigger modification if formation markers remain flat in two consecutive measurements
High-turnover mixed (both arms activated)	Formation markers (PINP ± BALP/OC) and resorption markers (β-CTX/NTX) are both elevated with large fluctuations	Use a balanced, safety-first combination of modules: moderate resorption-modulating + osteogenesis-supporting + anti-inflammatory/oxidative-stress buffering; avoid aggressive single-direction escalation	Rule out confounders (acute inflammation, hyperthyroidism, glucocorticoids); reassess fracture healing status and rehab plan	Recheck in 2–4 weeks; trigger review if variability persists or adverse events occur
Slow responder/stable low-turnover (minimal change)	Both formation and resorption markers change <∼10–15% over 8–12 weeks	Maintain baseline modules with conservative, stepwise refinement based on symptoms/function and tolerability; prioritize adherence and non-pharmacologic recovery supports	Confirm sampling timing/assay consistency; reassess goals; consider imaging/BMD schedule as appropriate	Recheck in 8–12 weeks; trigger earlier review if clinical deterioration or new risk factors emerge
Clinical–biomarker discordance	Pain/function trajectory inconsistent with BTM trend (improves but BTMs unfavorable, or *vice versa*)	Avoid “marker-only” escalation; conduct holistic reassessment (comorbidities, syndrome differentiation, confounders), and adjust modules only after confirming reproducible trends	Check confounders (renal function, recent diet/exercise, medications); repeat test; broaden labs if needed	Repeat BTMs within 1–2 weeks; trigger multidisciplinary review if discordance persists

^a^ Thresholds/time windows are illustrative and hypothesis-generating for decision-support within prospective protocols, not routine clinical recommendations. Threshold selection should be calibrated to assay characteristics, baseline variability, sampling timing, and patient-specific factors.

Biomarker-informed regimen optimization: under the TCM principle of “tonifying the kidneys and activating blood,” formula selection and modification could be aligned with the dominant remodeling state. For example, resorption-dominant patterns may prioritize strategies that restrain osteoclastogenesis and inflammation, whereas low-formation responses may emphasize pro-osteogenic and microenvironment-supporting components; blood-activating herbs may be considered to support microcirculation and the repair niche. Importantly, any adjustment should be implemented alongside standard postoperative care and safety monitoring.

### 4.2 Ethics, privacy, and data governance for real-time monitoring

The integration of high-frequency biomarker surveillance into clinical practice necessitates a preemptive strategy to navigate the attendant ethical and cybersecurity pitfalls that might otherwise jeopardize large-scale adoption. For the postoperative OVCF cohort—predominantly elderly patients susceptible to digital exclusion or cognitive barriers—these considerations are not merely peripheral but central to trial design. Consequently, a robust governance framework for biosensor-augmented workflows must be anchored by several non-negotiable pillars.

First, the paradigm of informed consent must evolve; it requires a transparent articulation of monitoring intensity and data-use trajectories, coupled with an uncomplicated “opt-out” pathway that safeguards the patient’s right to standard-of-care continuity. To prevent data bloat, minimization protocols should strictly limit collection to the essential physiological variables required for BTM interpretation. Furthermore, safeguarding patient identity demands a multi-layered defense: pseudonymization, robust encryption for data both in transit and at rest, and granular, audit-trailed access controls (Zhang W. et al., 2025; Wei et al., 2022; Cawthra et al., 2020; Jalali et al., 2021). From a jurisdictional standpoint, data localization must align with institutional mandates, strictly curbing secondary data exploitation without explicit re-consent. Crucially, the clinical accountability model must remain physician-centric; biosensor readouts function as adjunctive decision-support tools rather than autonomous diagnostic authorities, ensuring that the ultimate prescribing mandate resides with licensed clinicians.

Implementation feasibility may be enhanced by embedding monitoring into existing follow-up infrastructures—such as routine outpatient reviews—while maintaining kidney-tonifying and blood-activating formulas as a representative integrative option discussed in this review. This positioning frames the biosensor not as a rigid prerequisite, but as an enabling technology for precision medicine. By adopting a “minimum-necessary” data architecture—where raw identifiers remain sequestered within hospital-governed systems and only de-identified biomarker values are transmitted—multi-center deployment becomes pragmatic. Such a contained workflow ensures that continuous surveillance does not become an operational burden, reserving intensive review for instances where predefined safety thresholds are breached. To our knowledge, OVCF-specific prospective studies validating a biosensor-enabled BTM closed-loop workflow remain limited; therefore, the governance discussion herein adapts best practices from established remote patient monitoring and POCT ecosystems.

### 4.3 Challenges and future perspectives

Despite its transformative potential, the clinical trajectory of OPECT-driven management is hindered by systemic bottlenecks. To facilitate bench-to-bedside translation, technical refinements must prioritize long-term sensor stability and scalable, cost-effective manufacturing that aligns with routine clinical workflows. Beyond technical hurdles, the field necessitates a shift toward large-scale, multicenter prospective trials to generate high-level evidence—specifically targeting hard endpoints such as re-fracture rates.

The ultimate evolution of this paradigm, however, rests on the synthesis of unified diagnostic standards and integrated Chinese-Western clinical pathways. This requires deep-seated multidisciplinary synergy across orthopedics, endocrinology, and rehabilitative medicine. Future research should therefore concentrate on three convergent axes: the engineering of high-performance portable biosensing hardware, the execution of rigorous randomized controlled trials to validate cost-effectiveness, and the codification of consensus-driven clinical guidelines. By merging cutting-edge biotechnology with holistic medical perspectives, this framework offers a scalable, intelligent solution to the burgeoning public health challenge of OVCF in an aging global population.

## 5 Conclusion

The postoperative management of osteoporotic vertebral compression fracture (OVCF) is transitioning from predominantly “static intervention” toward more dynamic, metabolism-oriented regulation. This review discusses two major bottlenecks in current postoperative care: delayed assessment using BMD/imaging and limited feasibility of frequent BTM testing, as well as the challenge of tailoring systemic therapies to heterogeneous remodeling states. To address these gaps, we propose a closed-loop conceptual framework integrating OPECT-enabled biosensing for BTM monitoring with kidney-tonifying and blood-activating TCM formulas, referred to as “dynamic monitoring–precision intervention.”

The proposed framework emphasizes synergy between measurable pharmacodynamic assessment and mechanism-informed therapeutic optimization. OPECT-based technologies may increase the feasibility of decentralized BTM monitoring, while representative TCM formulas offer multi-target regulation of bone remodeling and the microenvironment. Together, these elements provide a rationale for a “monitoring–assessment–intervention–re-monitoring” feedback structure that could support future standardization of biomarker-guided integrative management. Nevertheless, technical validation, data governance, and prospective clinical studies will be essential before broad implementation can be considered.
